# Treatment of Tourette Syndrome with Cannabinoids

**DOI:** 10.3233/BEN-120276

**Published:** 2013

**Authors:** Kirsten R. Müller-Vahl

**Affiliations:** Clinic of PsychiatrySocialpsychiatry and PsychotherapyHannover Medical SchoolCarl-Neuberg-Str. 1D-30625 HannoverGermany

**Keywords:** Tourette syndrome, tics, cannabinoids, cannabis sativa, THC

## Abstract

Cannabinoids have been used for hundred of years for medical purposes. To day, the cannabinoid delta-9-tetrahydrocannabinol (THC) and the cannabis extract nabiximols are approved for the treatment of nausea, anorexia and spasticity, respectively. In Tourette syndrome (TS) several anecdotal reports provided evidence that marijuana might be effective not only in the suppression of tics, but also in the treatment of associated behavioural problems. At the present time there are only two controlled trials available investigating the effect of THC in the treatment of TS. Using both self and examiner rating scales, in both studies a significant tic reduction could be observed after treatment with THC compared to placebo, without causing significant adverse effects. Available data about the effect of THC on obsessive-compulsive symptoms are inconsistent. According to a recent Cochrane review on the efficacy of cannabinoids in TS, definite conclusions cannot be drawn, because longer trials including a larger number of patients are missing. Notwithstanding this appraisal, by many experts THC is recommended for the treatment of TS in adult patients, when first line treatments failed to improve the tics. In treatment resistant adult patients, therefore, treatment with THC should be taken into consideration.

## 1. Introduction

Although the therapeutic spectrum in the treatment of Tourette syndrome (TS) has expanded during the last years, there is still a substantial number of patients who is unsatisfied with well established treatment strategies either due to less efficacy or significant adverse effects. In addition, there is still no therapy known that is not only effective in the treatment of tics, but also improves associated behavioural disorders. In those patients who are impaired not only by their tics, but also by psychiatric comorbidities combined treatment with several drugs is often inevitable [1]. Therefore, new therapeutic strategies are desirable that are more effective, cause less adverse effects, and ideally improve not only tics, but also associated behavioural problems. Against this background, many patients with TS seek alternative or complementary medicine including special diets and nutritional supplements [2,3] as well as legal and illegal drugs such as nicotine, alcohol and cannabis sativa [4, 5].

## 2. Medical use of cannabinoids

Cannabis has been used for medical purposes in many cultures for hundreds of years, in particular, for the treatment of pain, spasms, asthma, insomnia, depression, and loss of appetite. In the first half of the 20th century, cannabis-based medication almost completely lost its acceptation, among other things, because it did not succeed to identify the chemical structure of the ingredients of *Cannabis sativa* L. This situation changed in the 1960s, after the exact chemical structure of delta-9-tetrahydrocannabinol (THC), the most psychoactive ingredient of cannabis sativa, could be determined. Research on the medical use of cannabinoids was further stimulated when it became clear that cannabinoids act through specific receptors: a predominantly in the central nervous system located CB1 receptor and a CB2 receptor that is expressed primarily by immune tissues. In 1992, the first specific ligand that binds to cannabinoid receptors could be identified. To date, five different endocannabi-

ISSN 0953-4180/13/$27.50 © 2013 – IOS Press and the authors. All rights reserved noids are known, among them the two most important anandamide (arachidonoylethanolamide) and 2-arachidonoylglycerol (2-AG). There is substantial evidence that endocannabinoids affect the activity of excitatory neurotransmitters such as glutamate as well as inhibitory transmitters such as GABA and glycine, but also of several monoamines such as dopamine, serotonin, noradrenaline, acetylcholine, and neuropeptides (for review see [6]).

To day, in many countries the cannabinoid THC (dronabinol, nabilone) and the cannabis extract nabiximols (Sativex®) – containing THC:canabidiol (CBD) = 1:1 – are approved for clinical use for the treatment of nausea and vomiting associated with cancer chemotherapy, anorexia in HIV/AIDS, and spasticity in multiple sclerosis, respectively. However, there is substantial evidence that cannabinoids are also effective in the treatment of other conditions such as neuropathic pain, spasms and movement disorders (for review see [7]).

## 3. Anecdotal reports

In 1988, it has been suggested for the first time that use of smoked marijuana might be effective in the treatment of tics and behavioural symptoms in patients with TS. In a case study, Sandyk and Awerbuch [8] reported on three 15–39-year-old male patients who experienced an improvement of their tics and premonitory urges when smoking 1/2 to 2 marijuana cigarettes per day. In addition, the patients felt an improvement of self-mutilatory behaviour, attention span, and hypersexuality. In 1993, Hemming and Yellowlees [9] described a single case of a 36-year-old man with TS who reported that he had been symptom free for more than one year when taking one "cone" of marijuana per night.

In 1998, Müller-Vahl et al. [10] used a standardized questionnaire to perform a survey about the use of cannabis sativa and its effects on tics and psychiatric comorbidities in a larger group of TS patients. Of 64 consecutive adults who were interviewed, 17 reported about prior use of marijuana. Of these, 14 (82%) patients experienced a reduction or complete remission of motor and vocal tics and an amelioration of premonitory urges, obsessive-compulsive behaviour (OCB), and attention deficit hyperactivity disorder (ADHD). None of these patients reported about serious adverse effects or a deterioration of symptoms while smoking marijuana.

## 4. Uncontrolled single case studies

Up to now, there are no controlled trials available investigating the effect of marijuana or a cannabis extract in TS. In Germany (and many other countries), until today the use of marijuana – even for medical purposes – is illegal. The cannabis extract nabiximols is available for medical use only for a few years. Thus, available clinical trials investigating the therapeutic effect of cannabinoids in TS used delta-9-tetrahydrocannabinol (THC), the most psychoactive ingredient of *cannabis sativa L.* However, it can be assumed that most clinical effects of cannabis sativa are caused by THC, although cannabis sativa contains more than 60 different cannabinoids.

In 1999, the effects of pure THC have been investigated in TS for the first time in a prospective open uncontrolled trial: a 25-year-old male patient was treated once with a single dose of 10 mg THC orally [11]. The patient reported that he had used marijuana (2–3 g per day) illegally for many years not only to reduce his tics, but also to improve behavioural problems including ADHD, OCB, anxiety, lack of impulse control, and self injurious behaviour, but stopped smoking marijuana 3 days before entering the study. Two hours after THC treatment the total tic severity score of the Tourette's Syndrome Global Scale (TSGS) [12] improved from 41 to 7 and coprolalia disappeared. No adverse effects occurred. Measuring cognitive functions neuropsychological tests showed improved signal detection, sustained attention, and reaction time after treatment. The patient felt not only a tic improvement of 70%, but also an amelioration in attention, impulse control, OCB, and premonitory urges.

In another open uncontrolled single case study investigating a 24-year-old female, a combined treatment with THC (10 mg/day) and amisulpride (1200 mg/day) was found superior compared to THC and amisulpride, respectively, alone [13]. Therefore, it can be speculated that THC augment anti-tic effects of dopamine receptor blocking drugs as suggested earlier by animal studies: In rats it has been demonstrating that haloperidol-induced hypokinesia significantly increases after co-administration of THC [14].

Brunnauer et al. [15] reported about a treatment resistant 42-year-old man whose tics decreased after treatment with 15 mg THC significantly (Yale Global Tic Severity Scale (YGTSS) [16] decreased from 89 to 22). Since he worked as a truck-driver, his driving ability was assessed using computerized tests to measure visual perception, reaction, concentration, and stress tolerance. Although the patient passed the test both in the drug-free phase and during THC treatment, his concentration and visual perception clearly improved after THC treatment. Thus, at least in patients with TS treatment with cannabinoids may result in improved driving ability.

A comparable observation has been made in a 28-year-old male suffering from ADHD (without tics) whose driving-related performance significantly improved after oral intake of THC [17]. The authors concluded that "... in persons with ADHD THC may have atypical and even performance-enhancing effects".

So far, there is only one single case report available describing the successful treatment of a 15-year-old boy with treatment refractory TS plus ADHD [18]. In this boy combined treatment with THC (up to 15 mg/day) plus aripiprazole (30 mg/day) and risperidone (3 mg/day) resulted not only in a marked tic reduction (YGTSS score decreased from 97 to 54), but also in an improvement in quality of life. No significant adverse effects occurred. Under THC treatment, for the first time, comedication with methylphenidate (30 mg/day) was well tolerated without tic exacerbation. Using transcranial magnetic stimulation (TMS), intracortical inhibition was found to be increased during THC treatment. The authors, therefore, suggested that THC might counteract deficits of intracortical inhibition in patients with TS and ADHD by modulating the release of several neurotransmitters including dopamine and F-aminobutyric acid.

## 5. Randomized controlled clinical trials

Until today, there are only two controlled trials available investigating the effects of orally administered pure THC in the treatment of TS. In a randomised double-blind placebo-controlled crossover single-dose trial 12 adult patients (11 men, 1 woman, mean age = 34 + 13 (SD) years, range, 18–66 years) were treated with 5, 7.5 or 10 mg THC (dosages were chosen according to patients' body weight, sex, age and prior use of marijuana) [19]. Patients were randomly assigned a single-dose of oral THC first or a single-dose of visually identical placebo first on two days separated by a 4-week washout phase. Using the self rating scale Tourette Syndrome Symptom List (TSSL) [20] a significant global tic improvement was found after THC compared with placebo ($p = 0.015$). In addition, a significant improvement of OCB ($p = 0.041$) could be assessed by TSSL. Using the examiner rating TSGS a significant improvement ($p = 0.015$) could be demonstrated for the subscore for complex motor tics. Data became more robust when including only those patients who had received 7.5 or 10.0 mg THC ($n = 8$) suggesting that higher dosages are more effective. All in all, 10/12 patients experienced a global improvement after THC (mean of + 35% ± 28.0, range, 20–90%), but only 3/12 after placebo (mean of + 7% ± 13.7, range, 10–40%).

In addition, the Symptom Checklist 90-R (SCL-90-R) [21] was used to evaluate different psychological symptoms. No influence of THC could be detected on depression, somatization, interpersonal sensitivity, anxiety, anger-hostility, paranoid ideation, and psychoticism, but there was evidence for a deterioration of OCB. From other studies, however, an improvement of OCB is suggested after treatment with cannabinoids [10,11].

No serious adverse reactions occurred. Five patients reported transient mild side effects after THC lasting between 1–6 hours (including headache, nausea, dizziness, hot flush, tiredness, poor powers of concentration, and cheerfulness). One patient reported dizziness, anxiety, tremble, sensitivity to noise and light, dry mouth, and ataxia after a single dose of 10 mg THC lasting half an hour.

In addition, a randomised double-blind parallel group placebo-controlled study over six-weeks has been performed including 24 adult patients with TS (19 men, 5 women, mean age = 33 + 11 (SD) years, range, 18–68 years) [22]. Starting at 2.5 mg THC/day the dosage was increased by 2.5 mg every four days to the target dosage of 10 mg. If a patient was unable to tolerate the maximum dose, the medication was reduced by up to 5.0 mg/day until a tolerated dose was achieved. The study consisted of 6 visits (visit 1 = baseline, visits 2–4 during treatment, visits 5 and 6 after withdrawal). Using the Global Clinical Impression Scale (GCIS) [20], at visits 3 and 4, respectively, a significant difference ($p < 0.05$) was found between the THC and placebo groups. Using ANOVA, there was a trend towards an overall significant difference ($p = 0.079$). Using the Shapiro Tourette Syndrome Severity Scale (STSS) [23], a significant group difference could be demonstrated ($p = 0.033$) at visit 4. At the same visit, both the subscore "motor global scale" of the YGTSS ($p = 0.040$) as well as the Rush videotape-based rating scale [24] ($p = 0.030$) demonstrated a significant difference between both groups. The self rating TSSL demonstrated a significant difference ($p < 0.05$) between the placebo and THC group on 10 treatment days (between day 16 and 41). Using ANOVA there was an overall significant difference between the two groups ($p = 0.037$). Several other measures, in addition, demonstrated a trend ($p < 0.1$).

Seven patients dropped out of the study, but only one due to adverse effects (anxiety and restlessness). No serious adverse effects occurred. Five patients in the THC and three in the placebo group reported mild side effects (tiredness, dry mouth, dizziness, muzziness, anxiety, and depression).

## 6. Effects of cannabinoids on neuropsychological performance

The above mentioned controlled trials aimed to investigate the effect of THC not only on tics and associated behavioural problems, but also on neuropsychological performance. In the single-dose cross-over study a variety of neuropsychological tests was performed, but no detrimental effects of THC could be detected on short-term verbal and visual memory, recognition, verbal learning, intelligence, information processing, vigilance, reaction time, sustained attention and divided attention [25].

In the six-week parallel group study the following tests were performed to investigate cognitive functions: German version of the Auditory Verbal Learning Test (VLMT) [26], Benton-Visual-Retention-Test (BVRT) [27], Divided Attention (TAP) [28], and Multiple choice vocabulary test (Mehrfachwahl-Wortschatztest, MWT-B) [29]. Altogether – neither during treatment nor after withdrawal – no detrimental effects were seen on learning curve, interference, recall and recognition of word lists, immediate visual memory span, and divided attention. Measuring immediate verbal memory span there was even a trend towards an improvement during and after treatment with THC [30].

These results are in line with the case report by Brunnauer et al. [15] describing a truck-driver whose concentration and visual perception improved after THC treatment, but in contrast compared to data obtained from healthy cannabis users. In healthy users it has been demonstrated that cannabis use may cause cognitive impairment [31]. Since it can be speculated that the central cannabinoid system might be involved in the pathophysiology of TS, it is conceivable that treatment of THC in patients with TS may result in different effects on neuropsychological performance compared to healthy cannabis users.

## 7. Averse effects and contraindications

Based on available studies and case reports it can be assumed that adverse effects in patients with TS do not differ from adverse effects described in other groups of patients. It can be assumed that cannabis, cannabis extracts (such as nabiximols) and individual cannabinoid receptor agonists (such as dronabinol and nabilone) show very similar or even identical side effects. Cannabinoids are generally considered as well-tolerated. The American Institute of Medicine declared that "Marijuana is not a completely benign substance. It is a powerful drug with a variety of effects. However, except for the harms associated with smoking, the adverse effects of marijuana use are within the range of effects tolerated for other medications" [32]. The most common side effects are tiredness and dizziness (in more than 10% of patients), psychological effects and dry mouth. Most commonly reported psychological effects are relaxation, euphoria, dysphoria, unpleasant feelings, heightened sensory and altered time perception, anxiety and panic (but also reduction of anxiety), impairment of memory, reductions in psychomotor and cognitive performance, and disorientation. Tolerance to these side effects nearly always develops within a short time. Most of the adverse effects can be prevented by slow and individual titration. In children and adolescents (but not in adults) there is substantial evidence that regular cannabis use at high doses may cause not only long-term effects on cognitive performance, but also doubles to risk of psychosis in vulnerable individuals [33,34]. Beside dry mouth other physical effects may occur such as tachycardia, orthostatic hypotension, reduced lacrimation, muscle relaxation, and increased appetite. Withdrawal symptoms are hardly ever a problem in the therapeutic setting [7].

Cannabinoids are contraindicated in patients suffering from a psychotic illness. THC should be used with caution in patients with a history of substance abuse, pregnant and breast feeding women, children $<$ 18 years, and patients with significant cardiac disorder and hepatitis C [7].

## 8. The role of the CB1 receptor system in the pathophysiology of TS

Based on the beneficial effect of cannabinoids in the treatment of tics, it can be speculated that the central CB1 receptor system might be involved in the pathophysiology of TS. This hypothesis is supported by the fact that the highest density of CB1 receptors is located in the basal ganglia, cerebellum, and hippocampus. In addition, there are several lines of evidence suggesting a complex interaction between the $CB_1$ receptor and the dopaminergic system. However, to date there is only one study available using the $CB_1$ antagonist [123I]AM281 (N-(Morpholin-4-yl)-1-(2,4-dichlorophenyl)-5-(4-[123I]iodophenyl)-4-methyl-1H-pyrazole-3-carboxamide) and single photon emission computed tomography (SPECT) to investigate central cannabinoid $CB_1$ receptors in six patients with TS before and after THC treatment [35]. Although a specific binding of [123I]AM281 to $CB_1$ receptors could be detected, due to lack of a control group, no statement could be given as to whether $CB_1$ receptor binding sites are pathologically changed in TS. There is no evidence suggesting that TS is caused by genetic variations of the central cannabinoid receptor (CNR1) gene [36].

## 9. Conclusion and practical aspects

Available data obtained from several single case studies and two small controlled trials consistently provide evidence for beneficial effects of cannabinoids in the treatment of tics in patients with TS. In addition, there is some weak evidence that cannabinoids may improve also associated behavioural problems such as OCB, attention span, impulsivity, and autoaggression. Since neuropsychological tests failed to demonstrate detrimental effects of THC on memory, reaction time, concentration, and attention [15,25,30], it can be assumed that beneficial effects in patients with TS are caused by specific effects rather than secondary mechanisms due to sedation or decreased general activity. Since CB1 receptors are not only highly located in those brain regions that are thought to be involved in TS pathology, but also have a complex interaction with the dopaminergic system, it can be speculated that beneficial effects in TS are mediated directly though the central CB1 receptor system.

Limitations of the available studies that have to be addressed are the small sample size, short treatment period, large number of multiple comparisons, fixed dose approach, and possible selection bias. The authors of a recent Cochrane review [37] argued that definite conclusions on the efficacy of cannabinoids in TS cannot be drawn, because longer trials including a large number of patients are missing. Notwithstanding this appraisal, by many experts [1,37] THC is recommended for the treatment of TS in adult patients, when first line treatments failed to improve the tics. Thus, in treatment resistant adult patients therapy with THC should be taken into consideration.

Treatment with THC should be started at a low dose of 2.5 or 5 mg/day and slowly up titrated to a daily dose of 10–20 mg according to efficacy and tolerability. THC should be used twice or three times daily. From unpublished data, there is limited evidence that the cannabis extract nabiximols (Sativex®) – containing THC:CBD = 1:1 – can be used for the treatment of tics, too. Because costs for treatment with both THC and nabiximols are high and – at least in Europe – health insurances often refuse to cover these costs in patients with TS (due to lack of approval), in Germany patients can apply to the public authorities for a special approval for the use of medicinal cannabis [7]. From open uncontrolled case studies in a limited number of patients, it can be assumed that treatment with medicinal cannabis is also effective in TS, and in some patients even better tolerated than THC alone. Longer trials with larger numbers of patients are necessary to further establish the efficacy and safety of different cannabinoids in the treatment of TS.
